# Neonatal Cullen's Sign

**Published:** 2014-04-01

**Authors:** Viroj Wiwanitkit

**Affiliations:** Visiting Professor, Hainan Medical University, China; visiting professor, Faculty of Medicine, University of Nis, Serbia; adjunct professor, Joseph Ayobabalola University, Nigeria

**Dear Sir,**

The recent report on Neonatal Cullen's Sign is very interesting [1]. Pederiva mentioned Neonatal Cullen's Sign as a distinguishing feature of intrauterine volvulus with hemorrhagic ascites.[1] Indeed, Cullen's Sign is a basic sign in medicine. It might imply the hemorrhage but it is not specific. Some non-hemorrhagic problems might also present Cullen's Sign. The good examples are the amebic infection [2] and cancer.[3] For intrauterine volvulus with hemorrhagic ascites, the diagnostic clue should be the ultrasonographic finding.[4,5]

## Footnotes

**Source of Support:** Nil

**Conflict of Interest:** None declared

